# Oral tongue cancer in public hospitals in Madrid, Spain (1990-2008)

**DOI:** 10.4317/medoral.21196

**Published:** 2016-10-01

**Authors:** Ana-Isabel García-Kass, Alicia Herrero-Sánchez, Germán Esparza-Gómez

**Affiliations:** 1 DDS, PhD, Department of Oral Medicine and Orofacial Surgery, School of Dentistry, Complutense University of Madrid; 2 MS, DDS, PhD, Professor in the Department of Oral Medicine and Orofacial Surgery, School of Dentistry, Complutense University of Madrid

## Abstract

**Background:**

The cancer which appears in the mobile portion of the tongue is the most common neoplasm of the oral cavity. The objective of this study was to analyse oral tongue cancer epidemiology in a population of 610 patients diagnosed between 1990 and 2008 and detailed in the Tumour Registry of the Madrid region.

**Material and Methods:**

A retrospective analysis based on the following variables provided in the Tumour Registry was achieved: age, gender, histology, stage, location, treatment. Descriptive and analytic statistics with these variables, using Pearson’s Chi-square test to study the relationship between the qualitative variables.

**Results:**

Patients’ mean age was 61.53±13.95 years, with a gender ratio of 2.09:1 (413 males vs 197 females). The lesion was mainly localized in the lateral border of tongue, with other sites (dorsal face, ventral face, lingual tonsil, contiguous sites, tongue NOS) represented at lower rates. Squamous cell carcinomas (94.9%) far outweighted other histologies (salivary gland tumours, soft tissue tumours, haematolymphoid tumours). 59% of the cases appeared in localized stages, versus 35.2% in regional and 4.8% in distant stages. Surgery was the most frequently used treatment, followed by surgery in combination with radiotherapy.

**Conclusions:**

Oral tongue cancer is a disease of the elderly, with a male predominance. It mainly appears in its lateral border, localized squamous cell carcinomas representing the great majority of lingual neoplasms.

**Key words:**Oral tongue cancer, squamous cell carcinoma, epidemiology, treatment.

## Introduction

Epidemiologists generally consider cancers of the tongue, gum, floor and other areas of mouth and pharynx as a unique group called oral cancer. However, some differences exist between these cancers in epidemiological terms, the most common neoplasm of the oral cavity being the cancer which appears in the mobile portion of the tongue (1). It exclusively constitutes the category of tongue cancer in clinical terms, as tumours of its base, which behave differently, are considered oropharynx tumours.

Although tongue cancer represents a health problem in certain countries of South-East Asia, where lately a significant increase in the incidence of the disease has been observed (2), an increasing number of incidents in Western countries such as the United States has also been detected.

Cancer deaths are reduced, first of all, through primary prevention - reducing incidence of the disease in the community - followed by better survival rates for patients through successful treatment. Therefore, all information concerning the survival of a patient diagnosed with cancer constitutes the most useful instrument for the control of the disease, together with the data of incidence and mortality (3). In this context, hospital and population-based cancer registries are extremely important. Although these registry systems are designed with different objectives and methodologies, they complement each other and are equally essential.

## Patient and Methods

The studied population consists of 610 patients diagnosed with tongue cancer between 1990 and 2008, coming from the Database of the Central Registry of Tumours of the Autonomous Community of Madrid. In this database, “case” is defined as every patient with a diagnosed tumour whose behaviour code (fifth digit according to the third edition of WHO’s International Classification of Diseases for Oncology, ICD-O-3) is equal or superior to 2, and whose topographic code is C02, thus malignant neoplasms of the base of the tongue remain excluded. Only primary tumours have been included. Three variables of age have been differentiated: “age in five-year periods”, “age in decades” and “under 65/65 or more years”. Tumour location and histology are codified according to the ICD-O-3. Due to the high number of histological groups given in the Tumour Registry, the cases have been pooled on the basis of the main categories, according to the three first digits of their ICD-O-3 codification. In order to avoid data dispersion they were finally aggregated, resulting in two main groups: “Squamous cell carcinomas” and “Other histologies”. The SEER (Surveillance, Epidemiology and End Results) Staging System has been used for tumour extension, considering for tongue cancer the following categories: localized (cancer confined to the mobile tongue regardless of size), regional (cancer extending to regional lymph nodes), distant (cancer spreading beyond the tongue or having involved tissues beyond those immediately draining the tongue) and unknown. In case of lymphomas, those located in a lymphatic region above or below the diaphragm were considered localized; those affecting more than one lymphatic region but at only one side of the diaphragm were considered regional, and those affecting lymphatic regions at both sides were designated distant. Surgery (with or without neck dissection), radiotherapy, chemotherapy, palliatives, surgery in combination with radiotherapy, chemotherapy with radiotherapy, surgery combined with chemo- and radiotherapy, and other treatments were the analysed therapeutic approaches.

The population has been described according to the aforementioned variables. The relationship between qualitative variables was analysed using Pearson’s Chi-square test, at a signification level of *p*<0.05. Data analysis was performed using the ‘SPSS for Windows’ statistics software, version 19.0 (Statistical Package for the Social Sciences, SPSS Inc., Chicago, IL). The study was approved by the Institutional Ethics Committee (C.I. 12/225-E).

## Results

* Population features

**Figure 1:**
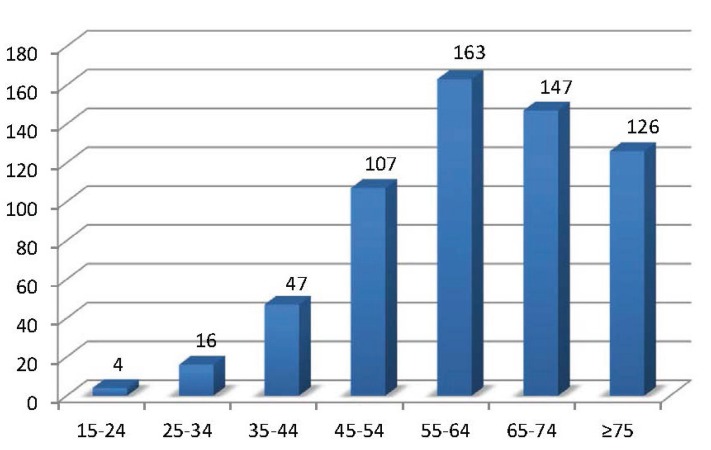
Diagnosed cases by age group in decades according to the age grouping established by SEER.

At the moment of diagnosis, patients were aged between 16 and 94 years, with a range of 77.88 years, 61.53±13.95 years being the mean age. When grouping the patients in five-year periods, we observe that most of the cases diagnosed are upwards from 45 years of age. The group of patients aged 60-64 years comprises the highest number of cases (14.9%). Figure 1 represents the distribution of patients by decades. 55.2% of the patients were younger than 65 years and 44.7% were 65 or older.

In our population, males were the most affected by the neoplasm (67.7%). The gender ratio was 2.09:1 (413 males vs 197 females). The mean age among males at the time of diagnosis was 60±12.9 years; among females this was 64.7±15.4 years. When analysing the distribution by gender and age, we observe that most diagnoses among males take place before the age of 65, while among females most take place from that age onwards, these differences being statistically significant (*p*<0.001).

**Figure 2:**
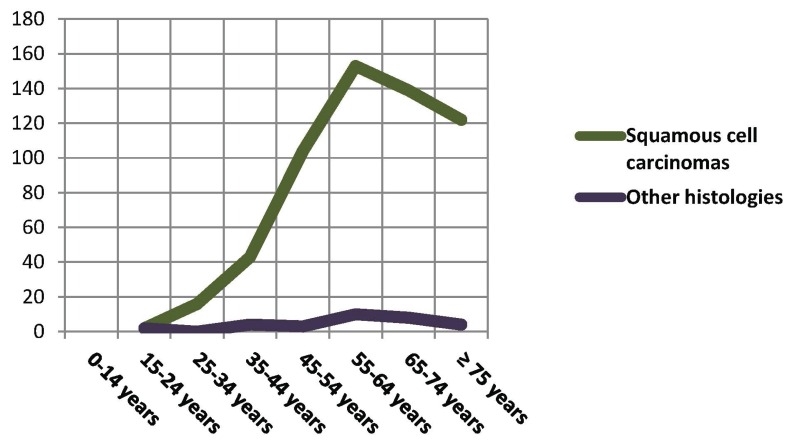
Distribution of frequencies of age groups in decades in each histological group.

* Tumour features

- Histology. Squamous cell carcinomas (SCC) represent the largest group, with 94.9% of the population. In  Table 1 the cases have been grouped according to similar histological features in accordance with the ICD-O-3 groups. SCCs stand out as the largest group, with a limited representation of the remaining neoplasms. For this reason they were pooled in a single category. For the statistical analysis the variable “grouped histologies” was used for the two most characteristic histological forms in this organ: squamous cell carcinomas (94.9%) and other histologies (5.1%). The mean age in diagnosis among patients with SCC was 61.6±13.8 years, very similar to the mean age of patients who presented other histologies (59.1±15.4 years) (*p*>0.05). The predominance of SCC is easily seen through a frequency distribution (Fig. 2). Thus, when evaluating the SCC evolution pattern, we observe that during the first decades of life its incidence is very limited. From 35 years on it starts to increase significantly, continuing up to 55-64 years, the age of maximum incidence. From that moment on it begins to decrease. In contrast, the other histologies show an undulating pattern which may be due to the complex collection of neoplasms in this group. Among males, SCCs represent the largest group with 386 cases (93.5%), compared with 27 cases of other histologies (6.5%). Among females, the differences between groups are more significant, with 193 squamous cell carcinomas (98%) versus only 4 cases (2%) of other histologies (*p*<0.05).

**Table 1:**
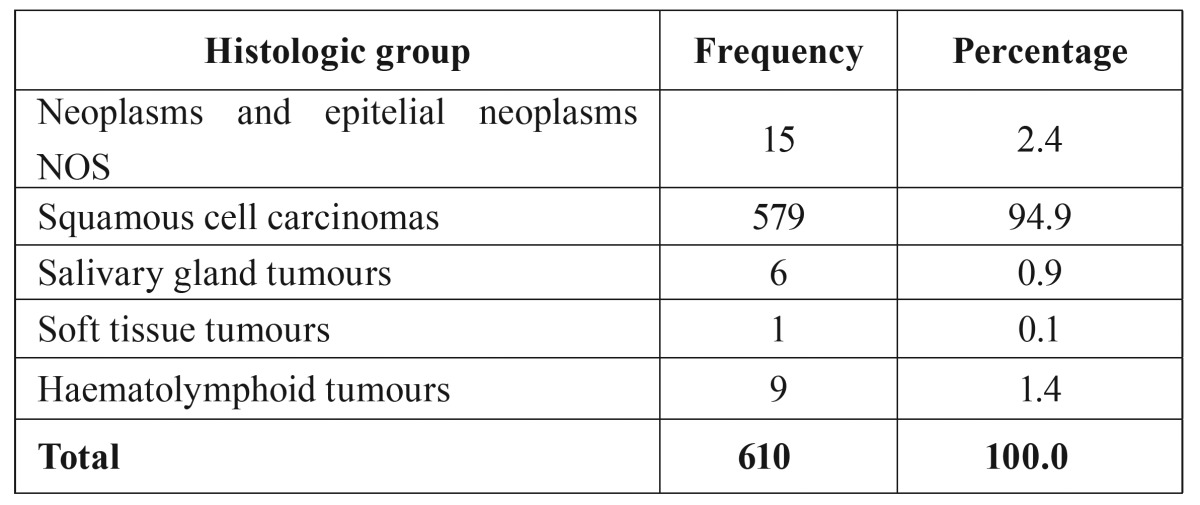
Distribution of cases according to histological groups.

**Figure 3:**
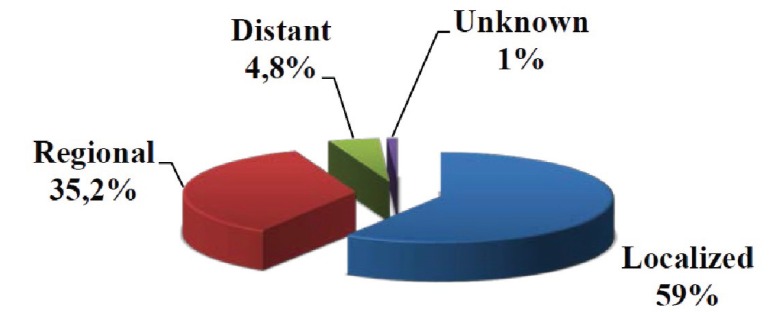
Distribution of the stage at diagnosis, in percentages.

- Tumour location. Tongue cancer is localized in the lateral border of this organ in 46.4% of cases, with the remaining sites represented at lower rates: dorsal face 6.1%, ventral face 4.9%, lingual tonsil 2.5%, contiguous sites 3.8%. However, an important proportion is represented by the NOS (Not Otherwise Specified) category (36.4%). When grouping the cases by location and gender, the observed pattern is maintained, with a higher number of cases for every location among males. We find important differences between ventral tongue and lingual tonsil in the gender ratio (5:1 - 25 males vs 5 females - and 14:1 - 14 males vs 1 female - respectively) (*p*<0.05). The topographic or anatomic distribution varies according to the type of tumour. Thus, 46.6% of SCCs are settled in the border of the tongue, 6.2% in the dorsal face, 5.2% in the ventral face, and 1.4% in the lingual tonsil. The remaining histologies were observed in the border of the tongue in 41.9% of occasions, 22.6% in lingual tonsil and 3.2% in the dorsal face (*p*<0.001). In almost every anatomical site the localized stage predominates. In ventral tongue, 73.3% of neoplasms are localized, 26.7% are regional and there are no distant ones. In the lateral border the localized (67.1%) and regional (29.7%) stages are predominant. However, the lingual tonsil does not follow this pattern, as the regional stages (46.7%) exceed the localized ones (40%), and a relatively high percentage of cases in distant stages (13.3%) is observed. In the dorsal face (37 cases) we find the following distribution: 59% localized, 35.1% regional and 2.7% distant (*p*<0.01). 

- Clinical stage of disease. At the time of diagnosis, 59% of the cases were in a localized and 35.2% a regional stage. In our population, only 4.8% of the tumours are distant (Fig. 3). Among younger patients, as well as among patients over 65, localized extension is the most frequent (54.9 vs 64%, respectively), followed by the regional and finally the distant extension. It should be noted that among patients under 65 years of age we find a higher percentage of cases in regional (39.2%, 132 cases) as well as distant stages (5.6%, 19 cases) when compared to the older patients (30.4% regional, 3.7% distant) (*p*<0.05). As the stage progresses, the gender ratio increases. In localized stages the gender ratio is 1.6:1 (225 males vs 135 females), in regional stages it is 3.05:1 (162 vs 53) and in the distant ones, 6.25:1 (25 vs 4) (*p*<0.001). This suggests that among women diagnosis is earlier. SCCs were mainly distributed in localized stages (58.9%), followed by regional (35.9%) stages, with only 4.1% presenting a distant stage.

Similarly, the other neoplasms (lymphomas, adenomas) are mainly diagnosed in localized (61.3%) and regional (22.6%) stages, with 16.1% in distant stages (*p*<0.05).

* Treatment features

The treatment options have been grouped according to the most frequent treatments, as described in Patients and methods, for the purpose of statistical analysis. The result is shown in  Table 2. The first treatment option was surgery in 77.9% of cases and radiotherapy in 10.5%. When a second treatment was needed (37.4% of cases), radiotherapy was the selected option in 27.5% of cases. A total of 297 cases were treated by isolated surgery, and 52 by isolated radiotherapy.

The distribution of the most common therapeutic approaches used as the first therapeutic option by the different variables can be seen in  Table 3. In the lingual tonsil, 26.7% of the tumours were treated with a combination of chemotherapy and radiotherapy. The remaining therapeutic approaches are found in lower proportions. It should be noted when analysing the distribution of tumour extensions in each therapeutic option, we observe that the patients surgically treated constitute the higher percentage of cases with localized extension (83.2%). Similarly, among patients treated with radiotherapy, the neoplasms in localized stages represent 67.3%. Patients who receive palliatives only present regional and distant stages. In the case of patients treated with chemotherapy, 28.6% were localized, 64.3% regional and 7.1% distant (*p*<0.001).

**Table 2:**
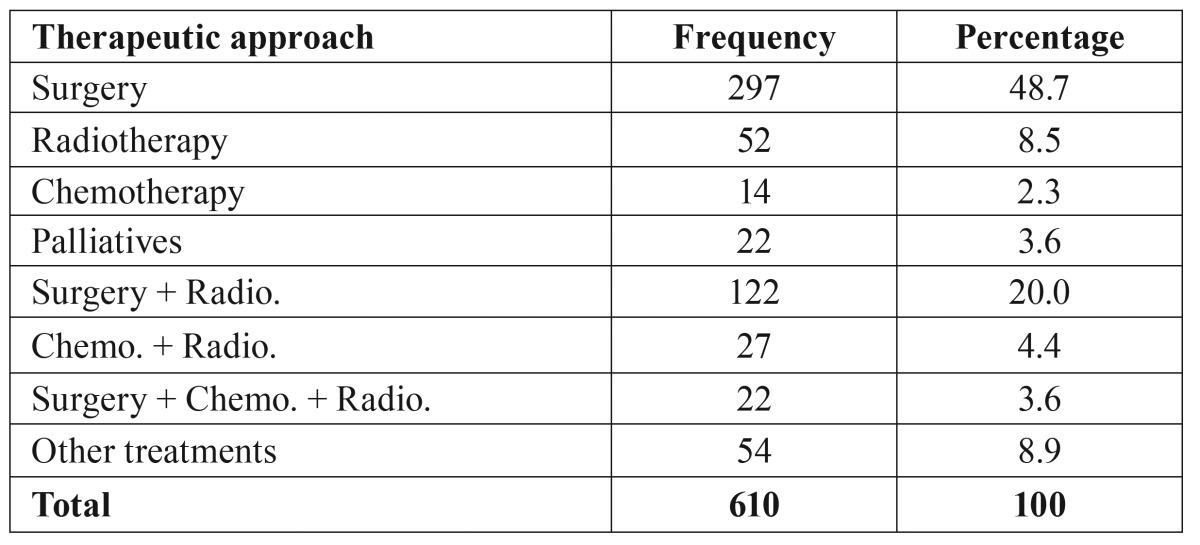
Therapeutic approach.

**Table 3:**
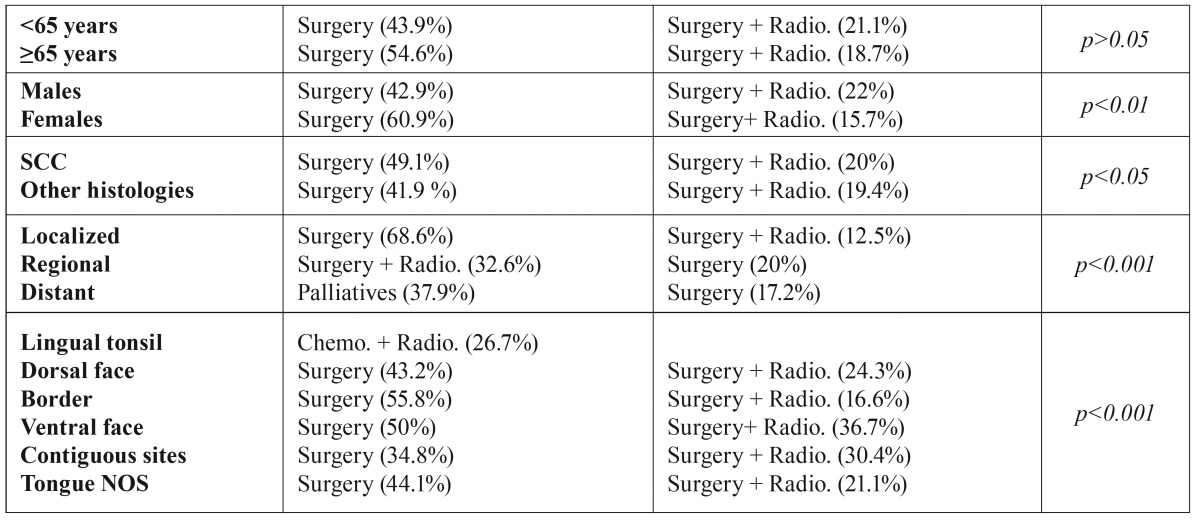
First therapeutic option according to studied variables.

## Discussion

The Central Tumour Registry of the Data Exchange System of the Autonomous Community of Madrid (Sistema de Intercambio de Datos de Cáncer, SIDC) is a hospital-based cancer registry. This means that every patient has been diagnosed and treated in hospital. Thus, there is a patient selection bias and it is not possible to calculate or predict incidences. These represent major limitations of our study. Although this does not affect the internal validity of the study, results are not transferable to the population. Moreover, variables enabling the estimation of risk factors such as tobacco, alcohol, traumatisms, candidiasis, nutritional deficiencies, family history, viral infections or poor oral hygiene are not registered. However, other information of interest, such as treatment, clinical follow-up (for survival studies, data not shown), histological diagnosis and extension, among others, is given by this type of registry.

SCC of the oral cavity is rare in patients under 50 years, being basically a disease which appears in the sixth or seventh decade of life. The mean age of our population with tongue cancer, 61.53±13.95 years, was very similar to that described in other series (4-8). However, in countries such as India, this mean age decreases to 49.4±11.9 years (9). The increase in incidence of tongue cancer among young males was first observed and reported during the 1980s in several analyses undertaken in the United States and Europe (10,11), suggesting the posterior evidence that this could be a global problem (12). Shiboski *et al.*, using the SEER database between 1973 and 2001 among white Americans aged 20 to 44 years, found a significant increase in SCC incidence, in the oral tongue as well as the base of the tongue and palatal tonsil. In the remaining oral and pharyngeal sites, its incidence decreased or remained constant (13). Due to the absence of habitual risk factors in certain cases of young patients, especially among women (14), it has been suggested that oral cancer among them could be a different disease than in older patients (15), with a different aetiology and progression, which acts in an aggressive way (16). However, the relatively anecdotal presence of these tumours in young adults and the diversity of criteria when selecting a cutoff point for age, location, stage and possible aetiology, complicate the comparisons between the different studies. Duroux *et al.* (17) consider that the singularity of oral cancer in patients under 40 years lies only in the early and massive character of alcohol-tobacco addiction. Thus, these patients could be more likely to reach pathological thresholds and develop a tumour of the oral cavity 10 or 20 years earlier than the rest of the population.

Most of the authors report a predominance of tongue cancer in men over women (7-9). However, evidence shows that this trend is reversing (4,5), probably due to behavioural changes among women, although strict regulations currently control public smoking in many countries, including most of Western Europe.

As in other oral sites, SCC represents the great majority of lingual neoplasms. In our series these neoplasms constitute 94.9%, a slightly larger percentage than that described by Izarzugaza *et al.* (91.7%), whose series included the base of tongue (18).

With regard to tumour extension, in Bombay 35.2% of anterior tongue cancers are diagnosed in early stages, 43.3% of them are regional and 4% distant stages (19). In contrast, the Finnish cancer registry reports that in 60% of mobile tongue cancers the neoplasm is present in localized stages, versus regional (18%) and distant (10%) ones. In the period under study a decrease in the proportion of tumours in localized stages is reported. This could be due to the fact that the definition of stages has changed in the last few decades. Thus, there is an observable increase in the number of cases where tumour extension is not specified, which could indicate that clinicians have become more careful when classifying a tumour as localized. This could result in an overestimation of the increase in survival in early cases, and an underestimation in the late ones (4).

When distinguishing between neoplasms affecting the anterior and basal areas of tongue, it has been reported that 56% of them involve the anterior area (7), where the probability of an earlier diagnosis is higher than in basal areas (8).

Haddadin *et al.* (7) and Herranz González-Botas *et al.* (6) regard surgery as the first therapeutic option, thus differing from other authors (8) who consider radiotherapy as the preferred therapy for the management of the primary lesion of the mobile tongue. Most of the studies in which the type of treatment by age is analysed, concentrating on young patients, use surgery as first therapeutic option (20). Manuel *et al.* combine surgery and radiotherapy, changing the order of both treatments. In the first instance, 36 of their 76 patients younger than 45 years with SCC of the mobile tongue were treated with radiotherapy, and the 40 remaining patients received surgery. The 36 patients initially treated with radiotherapy completed their treatment surgically, 23 of them to remove the residual tumour and 13 of them due to loco-regional failure. On the other hand, 24 of the 40 patients initially treated surgically received postoperative radiotherapy (21). Iype *et al.* use surgery and radiotherapy in equal proportions among their Indian patients younger than 35 years of age suffering from mobile tongue cancer. It should be noted that chemotherapy was used in 20.5% of the cases (22). We observe that histology significantly affects the type of treatment employed, as even though for both histological groups the preferred treatments were surgery and, in the second instance, surgery combined with radiotherapy, the “other histologies” group increases the proportion of chemotherapy, palliatives and chemotherapy plus radiotherapy. In our results, in line with other authors (8), we observe how the kind of treatment applied significantly depends on the tumour extension. Thus, localized tumours were basically surgically treated, while in the regional ones the combination of surgery and radiotherapy is chosen. As radiotherapy is considered a minimally invasive therapeutic procedure, it presents the advantage of preserving the shape and function of the tongue.

In conclusion, our population of 610 patients diagnosed between 1990 and 2008 with oral tongue cancer in Madrid, presented a mean age of 61.53±13.95 years, the gender ratio being 2.09:1. The lesion was mainly localized in the lateral border of the tongue, with predominantly squamous cell carcinomas. Most of the cases (59%) appeared in localized stages, versus 35.2% in regional and 4.8% in distant stages. Surgery was the most frequently used treatment, followed by surgery in combination with radiotherapy.
